# How Workers Live

**Published:** 1894-06-23

**Authors:** 


### Studies in Applied Sociology.

### XI.—HOW WORKERS LIVE—(*continued*).

It is sometimes said by those happy-go-lucky people who, as they say, trust in Providence, and leave Providence a good deal to look after, that a large family is no greater trouble nor expense than a small one. If asked to explain their meaning they would be rather at a loss, yet in their rough generalisation there is a certain truth. In large families the older ones look after the younger after a haphazard fashion, and all learn, from sheer lack of parental petting and guarding, to look after themselves. Then the younger children wear the outgrown clothes of their seniors, thus prolonging their day of usefulness; and at least the expense of a large family is not in direct proportion to its numbers. Indeed, inquiring among workers shows that the larger the family the less the proportionate expense. This is probably characteristic of many middle-class families, though when the income provides a convenient margin beyond the expense of maintaining husband and wife alone, each child is, as a rule, maintained by an increasing encroachment on this margin.

But where the whole income is spent before these extra claims arise, the expense of children can be met only by certain reductions in the average of comfort hitherto maintained. We find this even in that expenditure which can the least bear reduction—that on food. To find out the true proportion between size and expense in this matter, it is, however, necessary to form a basis for comparison by assuming a certain requirement for each member of the family. Taking the husband as the largest eater, we may set down his consumption at one hundred parts, the wife may be reckoned at ninety, and the same proportion for children at the age when they are growing fast, from eleven to fourteen. Children between the ages of seven to ten may be placed at seventy-five; their juniors between four and six at forty, and infants between one and three at fifteen. This may be regarded as an arbitrary division, yet when applied to a number of families it will give a fair idea of the relation between number and cost. Thus it has been noted that the expense for food of the average cotton operative in Britain, where the family consists only of husband and wife—*i.e.*, 190 units of consumption—is about £18 for every 100 units; but add a child, and you are forced to reduce the expenditure on every hundred units to £15. Another child takes off £2 more per hundred, and each successive one brings down the average till we find that in families with five children, implying with their varying ages, a total of 476 units of consumption, the average expenditure for each 100 units is only £10. In other words, while a husband and wife spend £36 on their own food, they will manage to feed five children besides on £47 10s. In the woollen industry the sums are smaller. A wool weaver and his wife spend £30 in food while they have no children, and £40 when they have five children to provide for. Glass-workers, who are generally better paid, do not observe the sequence so strictly, for we find with them that the first child usually causes increased expense, relatively as well as absolutely. Thus we find that the husband and wife average £37; the addition of one child brings up the expense to £41, an average of £1 per 100 units more. A second child brings about an absolute as well as relative reduction of the sum spent on food to £40 16s.; and further additions bring down the allowance till, instead of the £19 per 100 units they began with, the family consumption is reduced to £11 10s. per 100, or for the family £46 a year.

This might be conveniently used as an argument against large families. For every mouth that comes all must have a little less to eat. Carried on indefinitely, it must be admitted that the process leads to ultimate semi-starvation; but up to families of five, which we have taken as the extreme size of the normal family, no real fear of hunger need be looked for. But certain luxuries must be cut off—even the poor have their luxuries, and the family will be well or ill-fed according as the house-mother is or is not wise enough to buy food of the best nutritive value.

In different industries different foods preponderate. The textile worker allows on an average 95 lbs. of meat per annum for ever 100 units of consumption; in other words, the husband will get about ¼ lb. of meat a day throughout a year, and his wife and children less in the proportions we have stated; while steel workers indulge in 114 lbs. for every 100 units. On the other hand, the weaver consumes more flour than the steel worker—275 lbs. per 100 units instead of 208 lbs. He also takes about 7 lbs. of sugar more per 100 units, and a dozen more eggs; of butter, lard, and tea both take about the same, though both in tea and coffee the weaver a little exceeds the other. That is, in the calling which demands the greater muscular exertion, a greater amount of nitrogenous food is required. All workers in Britain are heavy consumers of meat, as compared with those of the Continent. Even in the iron industry the German with 96½ lbs. per 100 units is little more extravagant than the English weaver, while the Frenchman consumes only 57¾ lbs., and the Belgian 55½ lbs. On the other hand, the latter consume more flour and eggs, more than twice as much of each. Their consumption of coffee, too, is large—14 lbs. and 19 lbs. respectively to the Englishman's 3½ lbs.; but, on the other hand, tea does not appear in their accounts. It is almost needless to say that these quantities are all exceeded in America. The Illinois iron worker manages to consume 393 lbs. of meat per 100 units; and though this is excessive, we find 206½ lbs. put down as the average of Pennsylvania, 197¾ lbs. for Ohio, 187¼ lbs. for West Virginia, and 155 lbs. for Tennessee. The consumption of flour stands about 250 lbs. for the States in general, though greedy Illinois has 366 lbs.; and sugar, butter, and eggs are everywhere more lavishly used than in Europe. There, as here, however, we find workers in the iron and steel industries more self-indulgent than those of other trades, and the butchers' bills of the weaver cannot compare with those of the steel worker, though they are still far above the expenditure of even the most extravagant worker of Europe.

